# Role of endothelial CXCR4 in the development of aortic valve stenosis

**DOI:** 10.3389/fcvm.2022.971321

**Published:** 2022-09-06

**Authors:** Anna Winnicki, James Gadd, Vahagn Ohanyan, Gilbert Hernandez, Yang Wang, Molly Enrick, Hannah McKillen, Matthew Kiedrowski, Dipan Kundu, Karlina Kegecik, Marc Penn, William M. Chilian, Liya Yin, Feng Dong

**Affiliations:** ^1^Department of Integrative Medical Sciences, Northeast Ohio Medical University, Rootstown Township, OH, United States; ^2^Summa Cardiovascular Institute, Summa Health, Akron, OH, United States

**Keywords:** CXCR4, aortic stenosis, aortic valve stenosis, cardiac hypertrophy, endothelium

## Abstract

**Background:**

CXCL12/CXCR4 signaling is essential in cardiac development and repair, however, its contribution to aortic valve stenosis (AVS) remains unclear. In this study, we tested the role of endothelial CXCR4 on the development of AVS.

**Materials and methods:**

We generated CXCR4 endothelial cell-specific knockout mice (EC CXCR4 KO) by crossing CXCR4^fl/fl^ mice with Tie2-Cre mice to study the role of endothelial cell CXCR4 in AVS. CXCR4^fl/fl^ mice were used as controls. Echocardiography was used to assess the aortic valve and cardiac function. Heart samples containing the aortic valve were stained using Alizarin Red for detection of calcification. Masson’s trichrome staining was used for the detection of fibrosis. The apex of the heart samples was stained with wheat germ agglutinin (WGA) to visualize ventricular hypertrophy.

**Results:**

Compared with the control group, the deletion of CXCR4 in endothelial cells led to significantly increased aortic valve peak velocity and aortic valve peak pressure gradient, with decreased aortic valve area and ejection fraction. EC CXCR4 KO mice also developed cardiac hypertrophy as evidenced by increased diastolic and systolic left ventricle posterior wall thickness (LVPW), cardiac myocyte size, and heart weight (HW) to body weight (BW) ratio. Our data also confirmed increased microcalcifications, interstitial fibrosis, and thickened valvular leaflets of the EC CXCR4 KO mice.

**Conclusion:**

The data collected throughout this study suggest the deletion of CXCR4 in endothelial cells is linked to the development of aortic valve stenosis and left ventricular hypertrophy. The statistically significant parameters measured indicate that endothelial cell CXCR4 plays an important role in aortic valve development and function. We have compiled compelling evidence that EC CXCR4 KO mice can be used as a novel model for AVS.

## Introduction

Aortic valve stenosis (AVS), also known as aortic stenosis (AS), is the narrowing of the left ventricular outflow tract. It is among the most common valvular heart diseases and affects around 2–4% of the human population 65 years and older (1–3). As the human population rapidly ages, there is a marked increase in AVS worldwide (4). Patients with AVS are often asymptomatic for years before developing what is known as irreversible late-stage calcification, or calcific aortic disease (CAVD) (1). This advanced stage of valvular thickening and calcification can cause symptoms such as angina, syncope, dyspnea, and heart failure, being a significant cause of morbidity and mortality among the elderly population (2, 3). The morbidity rate of severe, symptomatic AVS is around 50% within 2 years of diagnosis (2). Risk factors such as bicuspid aortic valves (BAV), diabetes, mechanical injury, hypertension, maleness, smoking, and hypercholesterolemia all contribute to the development and progression of this disease (2, 3, 5, 6). It is important to note that AVS accounts for 3–6% of congenital heart defects in neonates and infants, often developing during the first trimester and evolving throughout gestation (7). With the only successful treatment option for AVS being surgical valve replacement, there is an urgent need to develop new target therapies (3).

CXCL12, also known as stromal cell-derived factor-1 (SDF-1), is a homeostatic chemokine expressed in many cell types throughout the body, especially in the presence of tissue damage (1, 8–10). Its receptor, CXCR4, is a G-protein coupled receptor expressed on the surface of cell types such as endothelial cells, platelets, neurons, and stem cells (9, 11, 12). Evidence has linked CXCR4 to biological processes such as stem cell recruitment, tissue regeneration, angiogenesis, tumor metastasis, cancer development and progression, CNS disease, and cardiovascular diseases (9, 11, 13). CXCR4 is upregulated under conditions of hypoxia, stress injury, and in damaged vascular tissues (10). Our previous studies have demonstrated the importance of the CXCL12/CXCR4 axis in myocardial repair (14, 15). The CXCL12/CXCR4 axis plays an important role in tissue repair (16) and inflammation (1, 17–19). However, the role of endothelial CXCR4 on the development of AVS remains unclear. In the current study, we provide evidence that the endothelial knockout of CXCR4 leads to the early onset of AVS.

## Materials and methods

### Animals

The animal work in this study was approved by the Institutional Animal Care and Use Committee (IACUC) of Northeast Ohio Medical University. Tie2-Cre mice (# 008863) were purchased from Jackson Laboratories. CXCR4^fl/fl^ mice were reported before (16). Endothelial specific CXCR4 knockout mice (Tie2-Cre/CXCR4^fl/fl^ mice) were generated by crossing the CXCR4^fl/fl^ mice with Tie2-Cre mice. The deletion of CXCR4 in endothelial cells was confirmed by western blot. Animals were housed in temperature-controlled conditions allowing food and water *ad libitum* in an American Association for Accreditation of Laboratory Animal Care–approved animal facility. All animal experiments performed complied with the NIH guidelines (Guide for the Care and Use of Laboratory Animals). For this study, the control group, referenced throughout as “Control,” are CXCR4^fl/fl^ mice. The experimental group, referenced throughout as “EC CXCR4 KO,” consisted of Tie2-Cre/CXCR4^fl/fl^ mice. Equal numbers of both males and females were used throughout the study.

### Genotyping

DNA samples used for genotyping were extracted from tissue samples using standard procedures. Briefly, tissue samples were collected from pups, aged 10 days. Ear clippings or tail clippings were placed in a 1.5 ml microcentrifuge tube and kept at −20^°^C until processed. Tissue was thawed and then digested overnight at 55^°^C in 675 μl DNA Extraction Buffer mixed with 25 μl proteinase K (Amresco, #97062-242). Samples were purified using 700 μl 25:24:1 Phenol/Chloroform/Isoamyl Alcohol (Fisher, # BP1752I-400), centrifuged at 14,000 rpm for 10 min, then the supernatant was saved. Further purification using Chloroform (B & J, #24263) was performed, following the previously listed steps. DNA was precipitated using equal volume of isopropanol (Fisher, #A451SK-4) and centrifuged (20 min at 14,000 rpm). DNA pellets were washed with 70% ethanol and resuspended in 1× TE Buffer (65–70 μl). PCR was set up using TAKARA polymerase kit (TAKARA, #RR0062) and Jackson Laboratories protocols (Table 1). Genotyping primer pairs are detailed in Table 1. PCR products were analyzed using gel electrophoresis on 3% agarose gels (MidSci, #BE-GCA-500, Fenton, MO, United States).

**TABLE 1:** PCR primers used.

Protocol # 28368		CXCR4-Fl primers
C-F	(5′-3′)	CAC TAC GCA TGA CTC GAA ATG
WT-lox C-F	(5′-3′)	GTG TGC GGT GGT ATC CAG C

**Protocol # 41502**		**Tie2-Cre primers**

WT forward	(5′-3′)	CTG TGA CCT GAG TGC CCA GT
Common	(5′-3′)	CCA CAC ACG TGC ACA TAT AGA
Mutant forward	(5′-3′)	GCG TTT AAG TAA TGG GAT GGT C

### Tissue harvest and fixation

Mice were euthanized and hearts were thoroughly perfused using cold 1× PBS (Sigma, #P4417, Burlington, MA, United States). The heart was removed, washed in 1× PBS, weighed, and sectioned into thirds using a heart matrice (Braintree, #BS-SS-H 5005, Chicago, IL, United States), to ensure the valves were not damaged. The apex and base were fixed in 10% Neutral Buffered Formalin (Fisher, #22-110-683) overnight at room temperature. The midsection of the heart was snap frozen and stored at −80^°^C for later use. Tissue samples were then fixed, processed, and 5 μm sections were prepared using a Leica microtome.

### Histology

All samples were stained using Hematoxylin and Eosin (H&E) for general morphology. Base samples containing the aortic valve were stained using Alizarin Red (Sigma, #A5533, Burlington, MA, United States) for detection of calcification. Masson’s trichrome (Scytek, #TRM-1, Logan, UT, United States) staining was used for the detection of fibrosis and excess collagen deposition. Images were obtained using the slide scanner (Olympus BX61VS, Webster, TX, United States) at 40× magnification. Quantification and calculations for calcification, collagen deposition, and leaflet thickness were performed using ImageJ software (NIH website).

### Immunohistochemical staining

Apex sample sections were stained with rhodamine-conjugated wheat germ agglutinin (WGA, Vector Laboratories, #RL1022), which labels myocyte membranes to visualize ventricular hypertrophy, as previously described (16). Images were acquired using a confocal microscope. All quantitative evaluations were performed with ImageJ software (NIH website).

### Echocardiography

Echocardiography was performed on mice aged 3–40 weeks under 1.5–2% isoflurane using the VEVO 770 machine. Left ventricular wall thickness, ejection fraction, and fractional shortening were calculated with VEVO LAB 3.0 software. Aortic velocity and pressure were measured *via* the echocardiogram and the aortic valve area was measured using the continuity equation.

### Endothelial cell isolation and culture

Mouse cardiac endothelial cells (ECs) were isolated as previously described (20). Briefly, mouse hearts were dissected and minced into small pieces. After the digestion of the heart using Collagenase I (Worthington, Lakewood, NJ, United States), cells were washed and incubated with Dynabeads conjugated with anti-CD31 antibody (Thermo Fisher Scientific, Oakwood, OH, United States). The beads with endothelial cells were washed several times and cultured in a mouse endothelial culture medium (Cell Biologics, Chicago, IL, United States). When confluent, cells were purified with Dynabeads conjugated with anti-Mouse CD102 (ICAM2) antibody.

### Western blot

Protein was isolated from endothelial cells with a RIPA Kit (Sigma Aldrich, R0278, Burlington, MA, United States) supplemented with protease and phosphatase inhibitors. Protein concentration was determined *via* BCA protein assay (Thermo Fisher Scientific, 23227, Oakwood, OH, United States) per manufacturer’s instructions. Protein lysates (40 μg/lane) were loaded for probing CXCR4 (1:500 dilution; Abcam, Ab181020, Waltham, MA, United States). Following primary antibody incubation (overnight at 4^°^C), blots were incubated (1 h at room temperature) with a mouse anti-rabbit IgG-HRP (1:3,000 dilution; Santa Cruz, SC-2357, Dallas, TX, United States). Immunoreactive bands were detected using a western blot imaging system (Cytiva, Amersham ImageQuant 800, Marlborough, MA, United States). GAPDH was used as a loading control (1:400 dilution; Millipore Sigma, MAB374, Burlington, MA, United States).

### Statistical analysis

Data are represented as mean ± SD. Statistical significance between the two groups was determined using a 2-tailed Student *t*-test. One- or two-way ANOVA was used for multiple comparisons where appropriate. A probability value of *P* ≤ 0.05 was used to establish statistical significance.

## Results

### Role of endothelial cell CXCR4 on the development of aortic valve stenosis

**FIGURE 1:**
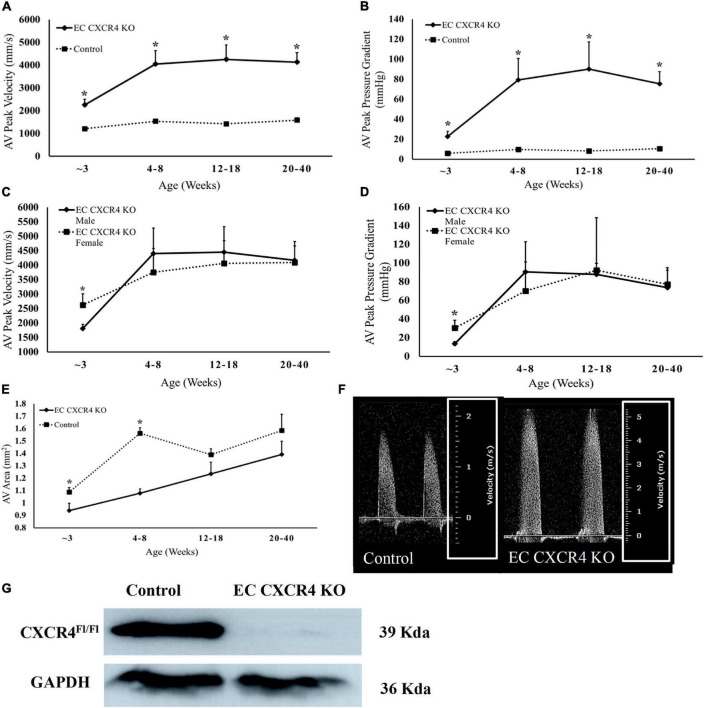
**(A–F)** Deletion of CXCR4 in endothelial cells led to Aortic Stenosis. **(A)** Aortic Valve Peak Velocity and **(B)** aortic peak pressure gradient (*n* = 10–13). **(C)** Aortic valve peak velocity and **(D)** aortic valve peak pressure gradient (*n* = 5–6), males and females are represented separately to show the difference in disease onset for EC CXCR4 KO mice. **(E)** Aortic valve area (*n* = 10–13) calculated using continuity equation. **(F)** Representative pulsed wave (PW) Doppler images of aortic flow for the control and EC CXCR4 KO groups, with images being from mice of the same sex and age. Note the different scales outlined in the white box. **(G)** Western Blot analysis of the CXCR4 knockout on endothelial cells. We observed a significant decrease in endothelial CXCR4 expression in EC CXCR4 KO mice compared with control mice. Data shows the average for mice aged 3–40 weeks. Calculated using Echocardiogram measurements on a VEVO 770 system. *Indicates a *p*-value ≤ 0.05 vs. control group.

To evaluate if endothelial cell CXCR4 deletion affects the function of aortic valves, echocardiography was performed on EC CXCR4 KO and control mice, spanning an age range of 3–40 weeks old. AV pressure gradient and velocities between the EC CXCR4 KO group and the control group were significantly different in different aged mice (Figures 1A,B). We observed that 50% of 3-week-old EC CXCR4 KO mice (*n* = 11) presented with significant increase in aortic valve peak velocity and aortic valve peak pressure gradient (Figures 1A,B,F). By 6 weeks old, 90% of EC CXCR4 KO mice (*n* = 11) presented with AVS of varying severity (Figures 1A,B). Forty-five percentage of 6-week-old EC CXCR4 KO mice developed severe AVS (aortic valve pressure gradient ≥ 40 mmHg, peak aortic velocity ≥ 4 m/s). Interestingly, our data also show significant difference in the aortic peak pressure gradients and velocities between the male and female EC CXCR4 KO mice at 3 weeks old (Figures 1C,D). However, such a sex significance was lost by 6 weeks old (Figures 1C,D). Moreover, the aortic valve area (AVA) of the EC CXCR4 KO mice was notably smaller than the control mice at 3–8 weeks old measurements yielded (*p* < 0.05, Figure 1E). The efficiency of knockdown of CXCR4 in endothelial cells was confirmed by western blot. A significant decrease in endothelial CXCR4 expression was observed in the EC CXCR4 KO mice compared with the control mice (Figure 1G).

### Role of endothelial cell CXCR4 on cardiac function and cardiac hypertrophy

**FIGURE 2:**
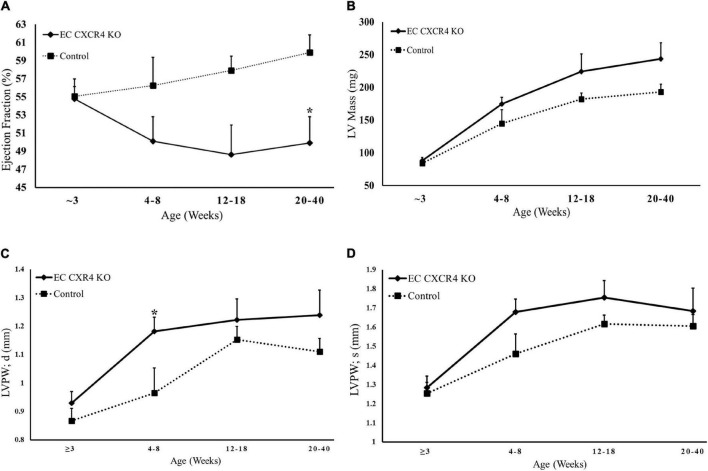
**(A–D)** Echocardiogram results indicate decreased Ejection Fraction and ventricular hypertrophy in EC CXCR4 KO mice. **(A)** Endocardial ejection fraction (B-mode) **(B)** Left ventricle mass **(C)** LVPWd, diastolic thicknesses of the LV posterior wall **(D)** LVPWs, systolic thicknesses of the LV posterior wall. Data shows the average for mice aged 3–40 weeks (*n* = 10–13). Calculated using echocardiogram measurements on a VEVO 770 system. *Indicates a *p*-value ≤ 0.05 vs. control group.

The EC CXCR4 KO mice have a significantly decreased ejection fraction (EF) compared to the control mice at the age of 20–40 weeks (Figure 2A). There was no statistically significant difference between the EC CXCR4 KO and control mice in the left ventricular mass (Figure 2B) or the left ventricle posterior wall systolic (LVPWs) although there was a trend (Figure 2D). Interestingly, the left ventricle posterior wall diastolic (LVPWd) between the EC CXCR4 KO and control mice was significantly different at the age of 4–8 weeks (Figure 2C). There was no significant difference in heart rate between the groups (data not shown).

**FIGURE 3:**
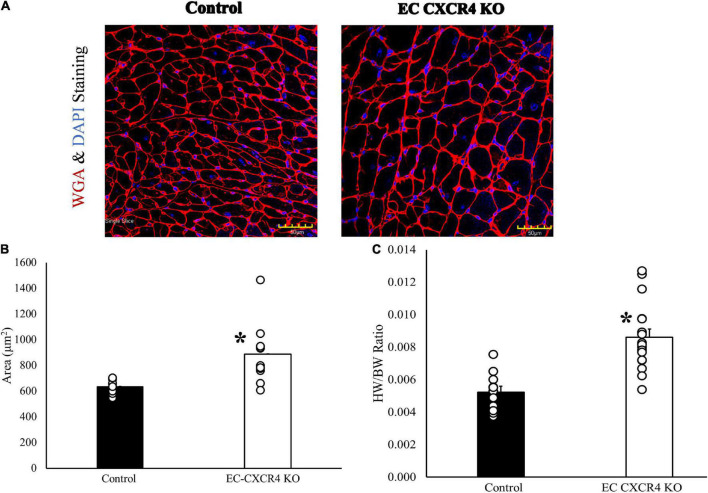
**(A–C)** Role of endothelial cell CXCR4 on cardiomyocyte hypertrophy. **(A)** Confocal image of representative immunofluorescent staining with WGA and DAPI to label myocyte membranes. **(B)** The average area of one cardiomyocyte was determined using WGA and DAPI staining (*n* = 10–12). **(C)** Heart weight/body weight ratio (*n* = 11–17). Our results showed a significantly increased cross-sectional area in cardiomyocytes and HW/BW ratio from EC CXCR4 KO mice compared to control mice. *Indicates a *p*-value ≤ 0.05 vs. control group.

We also evaluated the effects of endothelial cell CXCR4 KO on cardiac myocyte size with WGA immunostaining. Our results showed a significant increase in cross-sectional area of cardiomyocytes from EC CXCR4 KO mice compared to control mice (Figures 3A,B). We also found that the EC CXCR4 KO mice had a significantly larger heart weight (HW) to body weight (BW) ratio compared to the control group (Figure 3C), indicating enlarged heart, which is consistent with the immunofluorescence staining and the echocardiogram findings for LV hypertrophy.

### Endothelial cell CXCR4 KO led to increased microcalcifications, interstitial fibrosis, and thickened valvular leaflets

**FIGURE 4:**
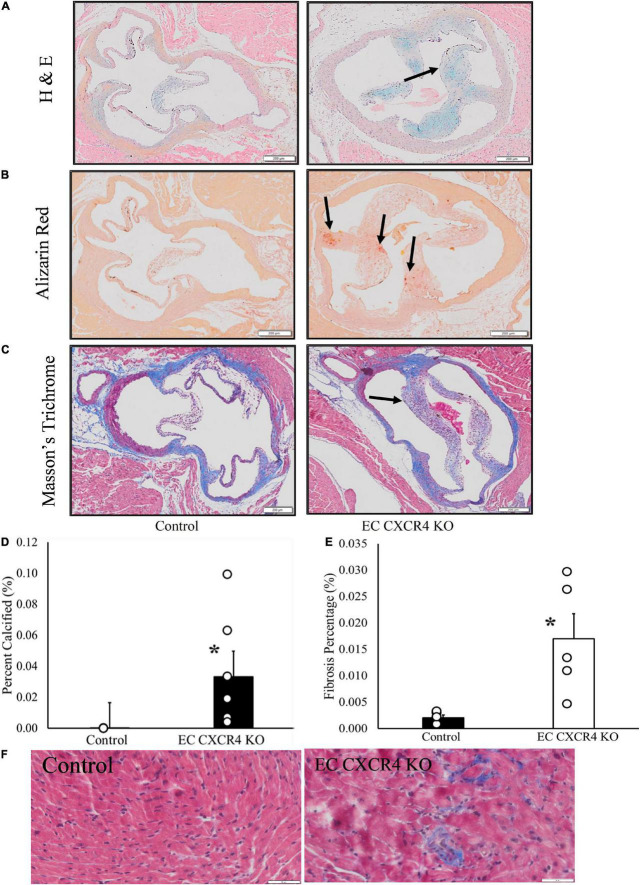
**(A–F)** Endothelial cell CXCR4 KO led to increased microcalcifications, interstitial fibrosis, and thickened valvular leaflets. **(A)** Modified Hematoxylin & Eosin staining. Arrows point to valve leaflet thickening. **(B)** Alizarin red staining. Arrows point to the positive stain results indicating the presence of microcalcifications. **(C)** Masson’s trichrome staining results. Arrow indicates the presence of leaflet thickening on aortic valves of the EC CXCR4 KO group. **(D)** Alizarin red staining quantification (*n* = 3–5). **(E)** Interstitial fibrosis quantification (*n* = 5). **(F)** Representative Masson’s trichrome staining images of interstitial fibrosis (scale bars = 50 μm). *Indicates a *p*-value ≤ 0.05 vs. control group.

The H&E staining and Masson’s trichrome staining show that valvular leaflets were thicker in the EC CXCR4 KO mice compared to the control mice (Figures 4A,C). Alizarin Red staining shows the deposition of calcium on the aortic valves of the EC CXCR4 KO mice (Figures 4B,D). Masson’s trichrome staining shows significant increase in interstitial fibrosis in the EC CXCR4 KO mice compared to the control groups (Figures 4E,F).

## Discussion

### Deletion of CXCR4 in endothelial cells linked to the development of aortic valve stenosis

In this study, we report the effect of endothelial CXCR4 expression on the development of AVS for the first time using our EC CXCR4 null mice. In human AVS, the aortic valve leaflets stiffen and are unable to fully open, causing significant increases in AV peak velocity and pressure gradient. This leads the heart to have to work harder to pump blood out to the rest of the body as the AV opening is narrowed. Therefore, as AVS develops and progresses, the pressure overload within the left ventricle increases, leading to an increase in left ventricle mass and eventual left ventricular hypertrophy. We showed a spontaneous murine model of AVS with significant increases in AV peak velocity and pressure gradient, decreases in AVA (Figure 1), and significant increases in valvular microcalcifications (Figure 4). Such a mouse model recaptures the pathology of AVS documented in humans, linking the absence of CXCR4 on endothelial cells to the development of hemodynamically stable AVS. Our EC CXCR4 KO mice show increased left ventricle posterior wall thickness in diastolic, enlarged cardiac myocyte size, and heart weight (HW) to body weight (BW) ratio, all of which indicate cardiomyocyte hypertrophy. Progression of the AVS and hypertrophy combined will cause restricted coronary flow, possibly leading to myocardial ischemia and fibrosis (3, 7).

Over the past few years, disruption of the CXCL12/CXCR4 pathway has been studied during embryonic development, using genetic models to knock out each respective piece of the axis to examine their effects on cardiac development and more specifically, valvular development (10, 21–23). Knockouts involving the CXCL12/CXCR4 axis are known to cause ventricular septal defects (VSDs), and developmental disruption of aortic arch, pulmonary artery, and coronary artery in animal models (10, 19, 21, 22, 24). CXCL12 null mice also present with malformations and decreased cardiac function (21). Hyperplasia within the semilunar valve (SLV) has been observed in CXCR4 knockout models, indicating the important role this receptor plays during the endothelial-mesenchymal transition and beyond (24). However, there has been very little done to analyze the AV function of adult KO mouse models (10, 21). To the best of our knowledge, this is the first study that links the deletion of CXCR4 in endothelial cells to the development of hemodynamically stable AVS in a murine model.

### EC CXCR4 KO mouse presents a new model for studying the development of aortic valve stenosis

Our Tie2-Cre driven, endothelial CXCR4 knockout mice show hemodynamically stable aortic valve stenosis, with calcification and ventricular hypertrophy. This genetic KO model develops AVS as early as 3 weeks in females and 6 weeks in males, maintaining increased AV peak velocities and pressures throughout their lifetime. Our mouse model of spontaneous AVS presents as a new avenue for AVS research, with a shorter development time compared to other AVS mouse models which normally take > 20 weeks to develop hemodynamically stable AVS (25, 26). Also, our AVS model does not need to feed the animals a special diet or induce a mechanical injury (25, 27). Even though about six varieties of dietary modification were used to develop AVS, they do not consistently develop hemodynamically significant AVS (25). Mechanical injury induced AVS models come with the risks associated with surgery (26). Our EC CXCR4 KO mice develop AVS early, presenting as a time frame friendly option to AVS without any forms of intervention.

### Male and female difference in disease progression

Human AVS has shown to progress differently in males and females. For example, males have a higher risk for the disease while females often present with more severe symptoms (28). Female patients often develop severe AVS with more fibrosis, but less calcification compared to male patients (4). It has been reported that estrogen may play a protective role against AVS, leading human females to develop the disease post-menopause (29). Also, there are sex differences observed in the development of AVS-related LV hypertrophy. Human males tend to develop eccentric hypertrophy while females often develop concentric hypertrophy (30). Therefore, understanding the relationship between AVS development and sex differences is important and to address this, we decided to include equal number of males and females in our initial study. Knowing these differences could cause some variation in both groups, we looked specifically at the male vs. female numbers at each time point. Interestingly, female EC CXCR4 KO mice show the signs of AVS as early as 3 weeks of age while the males did not present until after 5 weeks of age. Although the underlying mechanism is unclear, this may indicate that developmental hormones could play a role in the disease development. Further study such as the relationship between the CXCL12/CXCR4 axis and the different sex hormones is needed to address it.

### Possible mechanism and future directions

The goal of this study was to invest the role of endothelial CXCR4 on the development of AVS and establish the timeline for disease development. The mechanism to explain why AVS is occurring in the presence of an EC CXCR4 KO has not yet been determined. Previous research shows that CXCR4 is elevated in various organs at different stages in the developmental process, with CXCR4 KO mice presenting with defects in hematopoiesis, cardiogenesis, and fetal lethality *in utero* (31). CXCR4 is present on the endothelial cells that line both major vessels and the microvasculature throughout the body (32). This indicates that CXCL12/CXCR4 plays a key role in mediating cell migration and angiogenesis throughout development. Our findings that the endothelial-specific CXCR4 deficiency spontaneously developed AVS confirmed the critical role of CXCR4 in aortic valve development. It is possible that AVS developed in CXCR4 KO mice was due to congenital malformations.

Aortic valve stenosis may be brought on by multiple mechanisms, including mechanical injury and immune system activation (2, 3). CXCR4 signaling in blood vessels near calcified valves is speculated to be the cause of neovascularization and the promotion of inflammatory cell recruitment (13). Chemokines and their receptors are known for organizing and distributing immune responses throughout the body, so it is not surprising that the disruption of the CXCL12/CXCR4 pathway in any cell would lead to the development of diseases (9, 19, 32). AVS occurring in the EC CXCR4 KO mice may also be due to the role of CXCR4 in the recruitment of progenitor cells and immune-factor regulation as research has shown CXCR4 to impact endothelial progenitor cell migration and homing processes.

One limitation of our study pertains is the use of a Tie2-Cre for endothelial cell specific knockout. Although Tie2/Tek promoter are widely used in animal models that target endothelial cells, Tie2 may also express in hematopoietic cells (33), therefore, Tie2-Cre KO mice may affect the expression of CXCR4 in the hematopoietic cells. An inducible EC CXCR4 KO animal model will address the embryo developmental or postnatal contribution of CXCR4 to the AVS.

## Conclusion

We demonstrated that mice with deletion of CXCR4 in endothelial cells develop hemodynamically significant aortic valve stenosis and left ventricular hypertrophy. This indicates that CXCR4 plays an important role in aortic valve development and function. It was also observed that female mice in this line developed AVS earlier than males. Our results indicate that Tie2-Cre/CXCR4 ^fl/fl^ mice can be used as a novel model for AVS study.

## Data availability statement

The raw data supporting the conclusions of this article will be made available by the authors, without undue reservation.

## Ethics statement

The animal study was reviewed and approved by the Institutional Animal Care and Use Committee (IACUC) of Northeast Ohio Medical University.

## Author contributions

FD, MP, LY, and WC contributed to the conception and design of the study. AW, JG, VO, KK, and FD contributed to the echocardiography and data analysis. AW, FD, GH, MK, and HM contributed to the staining and data analysis. DK contributed to the EC cell culture and Western. LY, YW, AW, and ME contributed to the animal model breeding. AW organized the database and performed the statistical analysis. AW, LY, and FD wrote the manuscript. ME, VO, MK, DK, and HM contributed to the manuscript revision. All authors read and approved the submitted version.
